# The roles of metabolic pathways in maintaining primary dormancy of *Pinus koraiensis* seeds

**DOI:** 10.1186/s12870-019-2167-2

**Published:** 2019-12-11

**Authors:** Yuan Song, Jiaojun Zhu

**Affiliations:** 1 CAS Key Laboratory of Forest Ecology and Management, Institute of Applied Ecology, Shenyang, 110016 China; 2 Qingyuan Forest CERN, Chinese Academy of Sciences, Shenyang, 110016 China; 3 University of Chinese Academy of Sciences, Beijing, 100049 China

**Keywords:** Korean pine, primary dormancy, germination, metabonomics

## Abstract

**Background:**

Korean pine seeds have primary dormancy following dispersal, leading to poor seed germination and seedling establishment. Metabolic homeostasis determines whether the seeds are dormant or non-dormant. However, the specific metabolic pathways that maintain the primary dormancy of pine seeds are poorly understood.

**Results:**

Metabolic analysis was employed on the embryos of PDRS (seeds released from primary dormancy) and PDS (primary dormant seeds) on days 0, 5 and 11 after incubation under a germination-inductive temperature. A larger metabolic switch occurred in PDRS embryos from days 0 to 11. The contents of ninety metabolites were significantly changed from days 0 to 5, 83% of which (including most sugars, organic acids and amino acids) increased, reflecting that biosynthetic metabolism processes are initiated. The contents of ninety-two metabolites showed distinct variations from days 5 to 11, 71% of which (including most organic acids and almost all amino acids) reduced substantially. Fructose 6-phosphate, inositol-3-phosphate, 3-phosphoglyceric and D-glucose-6-phosphate contents showed the most decrease with decreasing 409-, 75-, 58- and 41-fold, indicating that the glycolysis and tricarboxylic acid (TCA) cycle strongly slowed down. The contents of the most metabolites in PDS embryos also displayed a relatively larger alteration only from days 0 to 5. Although 64% of metabolites increased from days 0 to 5, their levels were still lower compared with PDRS embryos. Furthermore, most metabolites were not further accumulated from days 5 to 11. Unlike PDRS embryos, almost all amino acids in PDS embryos did not exhibit a substantial decrease from days 5 to 11. Also, there was not a major decrease in the levels of metabolites involved mainly in glycolysis and TCA cycle, while some intermediates even increased.

**Conclusions:**

The attenuated biosynthetic metabolism processes, the lower utilization rate of amino acids and the higher operation rate of glycolysis and TCA in embryos maintain primary dormancy.

## Background

The MBKPFs (mixed broadleaf Korean pine (*Pinus koraiensis* Sieb. et Zucc.) forests), which were once dominant from Northeast China to the Far East region of Russia, have been decreasing in the past century because of large-scale industrial deforestation [1, 2]. MBKPFs have high plant diversity and play a vital, but poorly understood, role in ecosystem services (e.g., water conservation) due to a highly complicated structure [3]. Thus, it is becoming urgent to restore the degraded forests to the MBKPFs for forest management. Korean pine is the dominant tree species in MBKPFs, but their seeds have primary dormancy following dispersal and then enter secondary dormancy in the first summer after seed dispersal under natural conditions, thus leading to poor seed germination and seedling establishment [4–6]. It is essential to determine the primary dormancy mechanisms of Korean pine seeds to provide basis for recovering the MBKPFs.

Seed dormancy is defined as when an intact viable seed cannot complete germination when it is put under favorable conditions for germination [7]. As seed dormancy is gradually released, the range of conditions over which seeds complete germination progressively widens [8]. If the range of environments over which seeds complete germination cannot be increased, these seeds are nondormant [8, 9]. The germination process generally includes three phases: phase I is the process of rapid water absorption by the seed (imbibition), phase II is the process of reactivation of metabolism (lag), and phase III is the stage during which some part of the embryo protrudes from the seed coat [10]. By definition, germination sensu stricto starts with the uptake of water by the quiescent dry seed and terminates with the protrusion of the radicle and the elongation of the embryonic axis [10].

Metabolism is initiated to produce energy and build blocks for the various cellular processes of driving seed germination [10–12]. The differential proteomic analysis between dormant and nondormant seeds suggests that energy metabolism and protein metabolism play potential roles in promoting seed germination. For example, 25 proteins exhibited a differential accumulation pattern between dormant and nondormant *Arabidopsis* seeds after 1 day of imbibition [13]. Furthermore, one out of 8 high-abundance proteins in nondormant seeds is related to energy metabolism. 90% of down-regulated proteins in nondormant *Arabidopsis* seeds treated with ABA is involved mainly in energy and protein metabolism [13].

A block in metabolic pathways may invoke seed dormancy [14–16]. Metabolomics can detect the differential accumulation of various metabolites at the global level [17, 18]. Information is accumulating on the importance of metabolic pathways as a mechanism of seed dormancy of many herbaceous plants. For example, sucrose metabolism [18, 19], energy metabolism [20], lipid metabolism [13] and amino acid metabolism [18, 21] are repressed in the imbibed dormant seeds.

The potential of imbibed dormant seeds to synthesize protein [22–24] and produce ATP (adenosine triphosphate) [25] is lower following transfer of seeds to germination conditions. However, some studies revealed that the generation of sugar and amino acids is not inhibited in the imbibed dormant seeds of *Picea glauca* [14] and *Julans regia* [26] relative to moist chilled seeds. In the above-mentioned studies, the metabolic changes occurring during germination sensu stricto of nondormant seeds has not yet been determined. Therefore, the actual metabolic pathways maintaining seed dormancy might have been obscured because there is lack of the comparison between dormant and nondormant seeds under the same conditions.

There are only two studies focusing on the metabolic mechanism of seed dormancy in Korean pine. It has been reported that the seed dormancy of Korean pine is related to the lower levels of reducing sugars [27]. It can be found that lower respiratory metabolism may maintain the seed dormancy of Korean pine by comparing the respiration rates of dormant and nondormant seeds during the period of germination sensu stricto [28]. However, it is still unclear which metabolic pathway is changed to maintain seed dormancy. *Pinus koraiensis* seed has thick and hard seed coat, and the total thickness of seed coat varies between 1.20 and 1.30 cm [29]. The thickness of the dense stone cell layer of seed coat is between 0.40 and 0.43 cm, and the apparently thickened stone cell wall is visible [29]. Many seeds have thick seed coats that can impose dormancy by limiting the availability of oxygen to the embryo, inhibiting the water uptake, preventing the exit of inhibitors from the embryo, or applying mechanical restraint [30]. The previous works have showed that the application of respiration inhibitors such as cyanide and malonate (inhibit terminal oxidation reactions and TCA (tricarboxylic acid cycle) and fluoride (inhibits glycolysis) releases the seed dormancy of several species [30, 31]. When dormant sunflower seeds were imbibed at non-permissive temperatures for germination, TCA cycle and glycolysis were more active [20]. It can be inferred that seed coat interferes with gas exchange, imposing the hypoxia condition inside the seed. Concomitantly, the dormant seed consumes a large amount of oxygen through glycolysis and TCA cycle. Therefore, other aerobic processes cannot be normally performed, resulting in the maintenance of seed dormancy. This may be a reason for the maintenance of dormancy of hard seed. However, to our knowledge, there have been no studies conducted to document the relationship between the metabolic pathways, especially energy metabolisms, under hypoxia conditions and the maintenance of primary dormancy of pine seeds. Therefore, we test the hypothesis whether the specific alterations of some metabolic pathways maintain the primary dormancy of Korean pine seeds or not. The objective of our work is to fully understand the seed dormancy mechanism of Korean pine by providing a wide overview of alterations in primary metabolic processes occurring during germination sensu stricto of dormant and nondormant seeds. The inferences from this study will advance improve the understanding of seed dormancy mechanism of woody plants, and thus help establish a simple and very efficient method to release seed dormancy.

## Results

### Changes in the respiration rates of intact PDS and the PDS with cracked seed coats

**Fig. 1:**
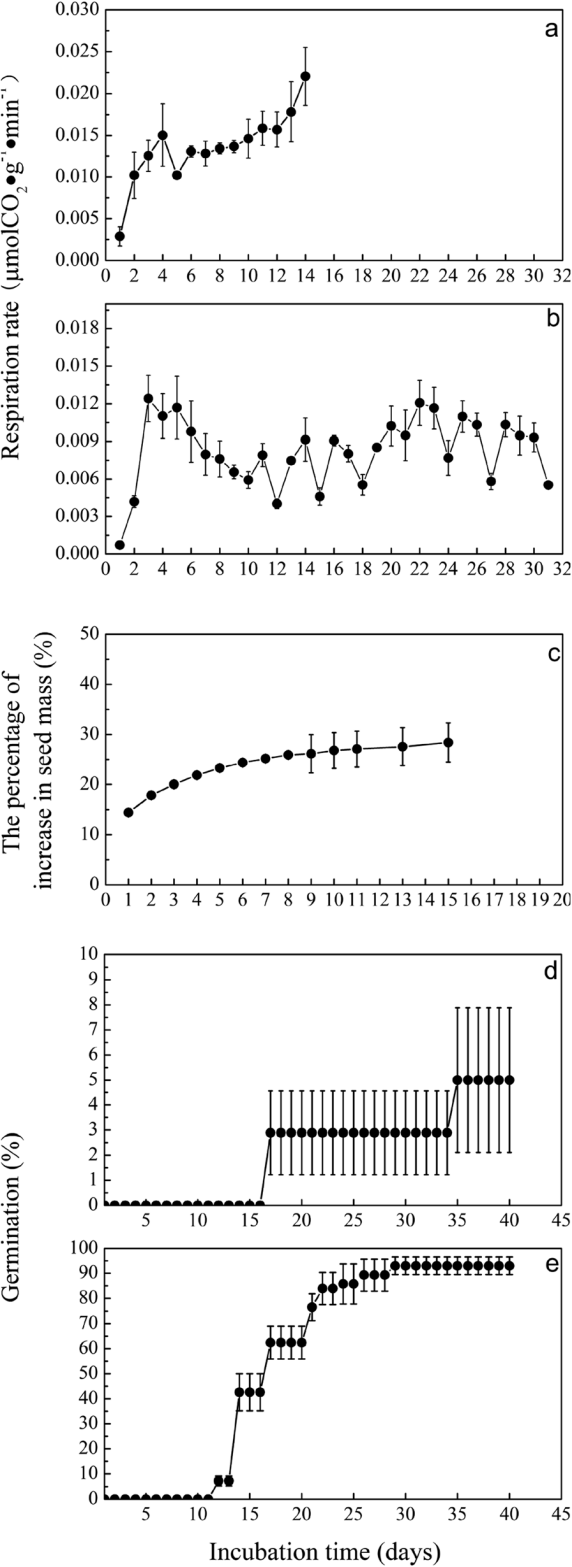
**a** Respiration rates of intact primary dormant seeds with cracked seed coats. **b** Respiration rates of intact primary dormant seeds. **c** Changes in the masses of intact primary dormant seeds**. d** Germination of primary dormancy released seeds. **e** Germination of primary dormant seeds

After seed coats were cracked, the respiratory rate of PDS significantly (*P* < 0.05) increased to 0.012 μmol CO_2_ g^− 1^ min^− 1^ on the fourth day of incubation compared with the first day of incubation (Fig. 1a). Furthermore, from the sixth day of incubation, the respiration rate continually increased and reached significantly (*P* < 0.05) a maximum (0.022 μmol CO_2_ g^− 1^ min^− 1^) on the fourteenth day of incubation when the germination is completed.

The respiratory rate of intact primary dormant seeds significantly (*P* < 0.05) increased to 0.012 μmol CO_2_ g^− 1^ min^− 1^ after 3 days of incubation and then gradually reduced to 0.006 μmol CO_2_ g^− 1^ min^− 1^ after 10 days of incubation (Fig. 1b). During the rest of time of incubation period, the respiratory rate varied between 0.004 and 0.012 μmol CO_2_ g^− 1^ min^− 1^.

### Changes in the masses of PDS

The masses of PDS rapidly significantly (*P* < 0.05) increased 23% on the fourth day of incubation compared with the first day of incubation and then slowly increased, varying between 22 and 28% during the rest of time of incubation period (Fig. 1c).

### Germination of PDRS and PDS

The germination percentage of PDRS was 93% (Fig. 1d), which was significantly higher than that of PDS (5%, *P* < 0.05) (Fig. 1e).

### Principal component analysis of metabolites in the embryos

**Fig. 2:**
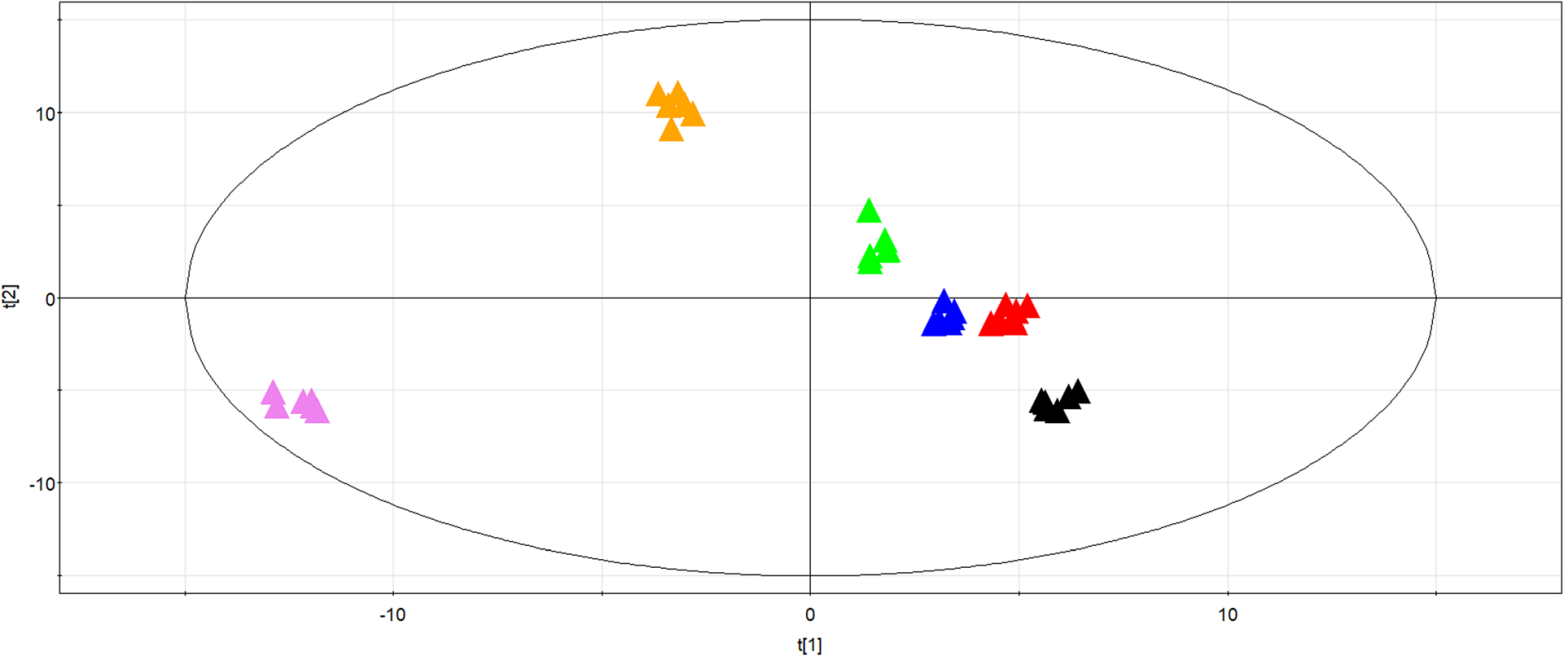
PCA score plot of principal component 1 and principal component 2 of the metabolite profile differentiating embryos of six different types of seeds, PDS (black triangle), PDS5 (red triangle), PDS11 (blue triangle), PDRS (green triangle), PDRS5 (orange triangle) and PDRS11 (violet triangle). PDRS, seeds released from primary dormancy; PDRS5, 5-day incubated seeds released from primary dormancy; PDRS11, 11-day incubated seeds released from primary dormancy; PDS, primary dormant seeds; PDS5, 5-day incubated primary dormant seeds and PDS11, 11-day incubated primary dormant seeds

PCA score plot is shown in Fig. 2, respectively. According to the PCA analysis, 72.4% of the total variance was explained by PC1 (40.6%) and PC2 (31.8%) (Fig. 2). The parameters of the model were as follows: R^2^X = 0.994, Q^2^ = 0.972. A clear formation of six distinct groups (PDRS, PDRS5, PDRS11, PDS, PDS5 and PDS11) was observed in the PCA score plot (Fig. 2). Both the PDRS5 and PDRS11 samples were discriminated from the PDRS along the first principal component. The maximum separation occurred between the PDRS5 and PDRS11 groups along the first principal component. The PDS samples were clearly grouped away from the PDS5 and PDS11 samples based on the second principal component. The PDS5 and PDS11 samples were situated relatively close to one another.

### Clustering analysis of metabolites in the embryos

**Fig. 3:**
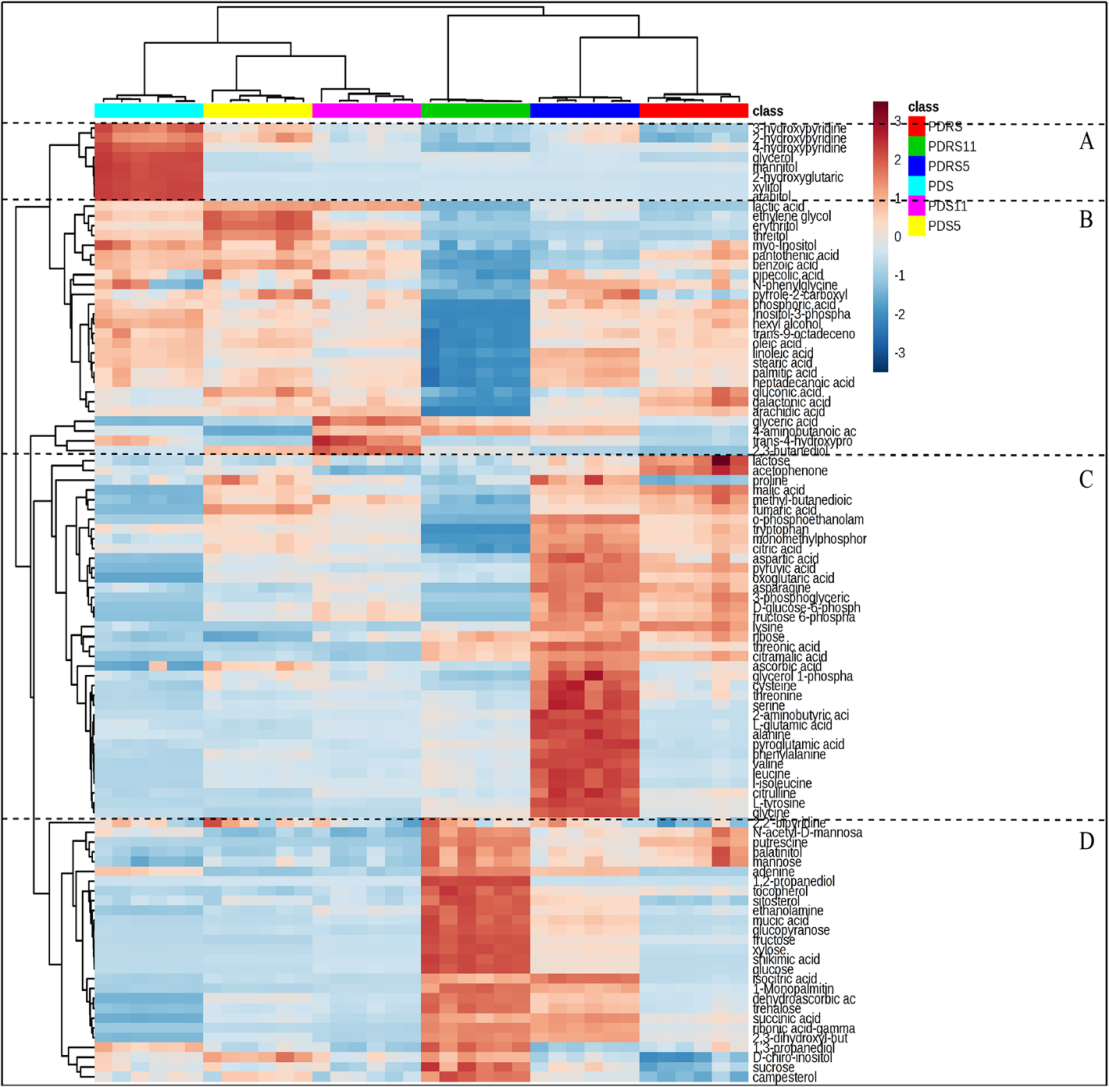
Heatmap of identified metabolites in the embryos of six different types of seeds. PDRS, seeds released from primary dormancy; PDRS5, 5-day incubated seeds released from primary dormancy; PDRS11, 11-day incubated seeds released from primary dormancy; PDS, primary dormant seeds; PDS5, 5-day incubated primary dormant seeds and PDS11, 11-day incubated primary dormant seeds

The HCA of identified metabolites was constructed to visualize the clustering of 98 differential components (depicted on the heat map, Fig. 3). Pearson correlation coefficients among differential metabolites were considered as a metric method to visualize the distance between metabolites. To determine the relationships and trends among the differential metabolites, a HCA plot were divided into four groups. In zone A, the contents of metabolites in group A (e.g., glycerol, mannitol, xylitol and arabitol) were the lowest in the embryos of PDS. Most of metabolites in group B exhibited the highest contents in the embryos of PDS, PDS5 and PDS11, which mainly contained polyols (e.g., ethylene glycol, erythritol, threitol, myo-inositol, inositol-3-phosphate, hexyl alcohol and 2,3-butanediol) and fatty acids (e.g., trans-9-octadecenoic acid, oleic acid, linoleic acid, stearic acid, palmitic acid, heptadecanoic acid and arachidic acid). A large proportion of amino acids, TCA cycle intermediates (e.g., malic acid, pyruvic acid, citric acid and oxoglutaric acid) and glycolysis cycle intermediates (e.g., D-glucose-6-phosphate, fructose-6-phosphate and 3-phosphoglyceric) clustered in group C. These metabolites had the highest contents in the embryos of PDRS5. The contents of the corresponding metabolites from group D were the highest in the embryos of PDRS11, including monosaccharides (e.g., mannose, trehalose, sucrose, fructose, glucopyranose, glucose and xylose). A relatively small change in the contents of these metabolites clustered in groups C and D were observed between the embryos of PDS, PDS5 and PDS11.

### Partial least squares discriminant analysis of metabolites in PDRS, PDRS5 and PDRS11 embryos

**Fig. 4:**
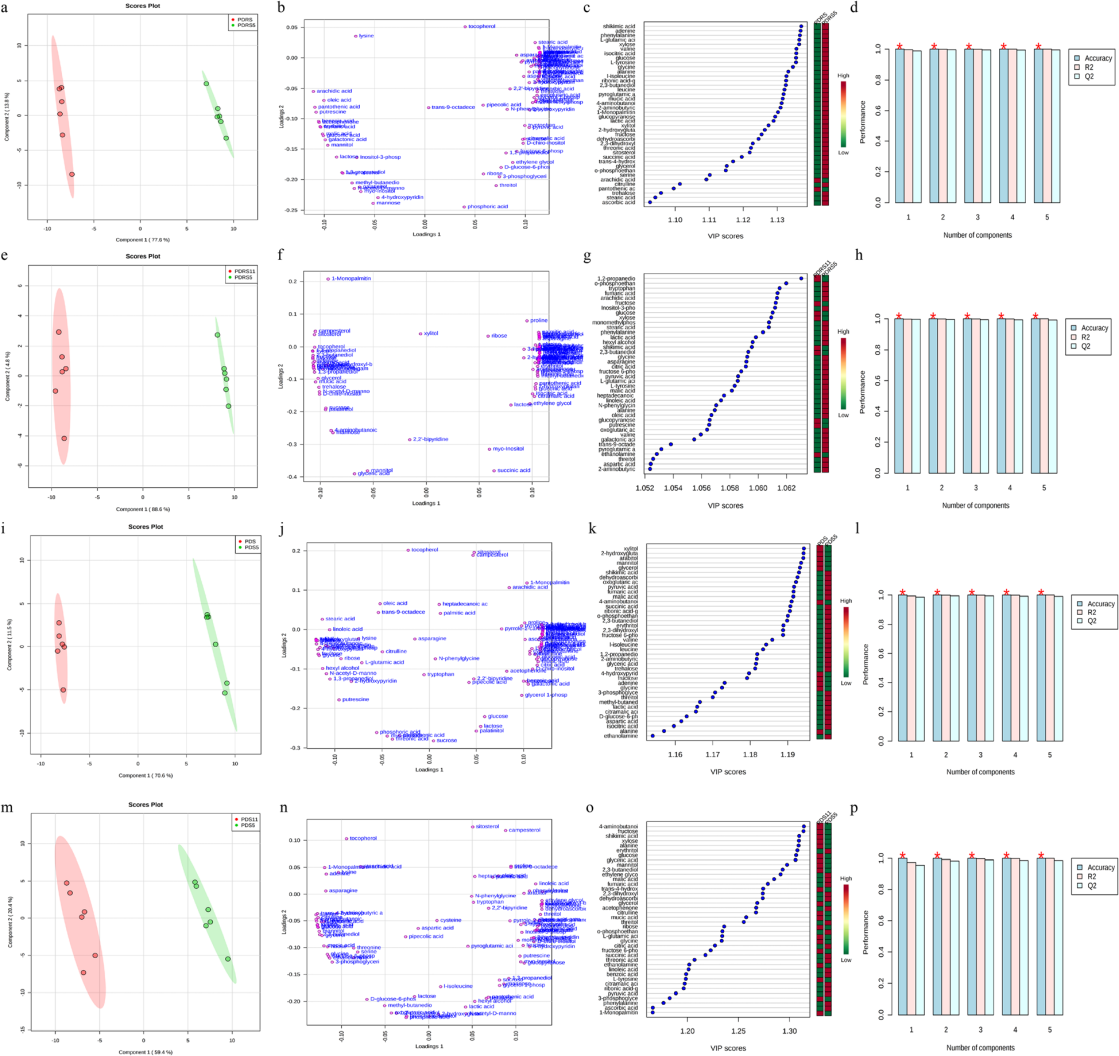
PLS-DA score plot of component 1 and component 2 of the metabolite profile differentiating the embryos of PDRS5 and PDRS (**a**), PDRS11 and PDRS5 (**e**), PDS5 and PDS (**i**), PDS11 and PDS5 (**m**). Metabolite loadings of components 1 and 2 showing the primary features responsible for differences between PDRS5 and PDRS (**b**), PDRS11 and PDRS5 (**f**), PDS5 and PDS (**j**), PDS11 and PDS5 (**n**). VIP score plots of top 40 metabolites contributing to metabolomic profile change for PDRS5 and PDRS (**c**), PDRS11 and PDRS5 (**g**), PDS5 and PDS (**k**), PDS11 and PDS5 (**o**). A 10-fold cross-validation performance model of the PLS-DA components built for origin differentiation of PDRS5 and PDRS (**d**), PDRS11 and PDRS5 (**h**), PDS5 and PDS (**l**), PDS11 and PDS5 (**p**)

The differences between PDRS5 and PDRS were mostly explained by PC1 (77.6%) (Fig. 4a). The R^2^ and Q^2^ values of the PLS-DA model were 0.99 and 0.98, respectively (Fig. 4d). Of the identified metabolites, ninety exhibited significant differences between PDRS5 and PDRS groups (*P* < 0.05). Sixty-five metabolites with VIP > 1 significantly contributed to the separation of PDRS5 from PDRS (Fig. 4b,c).

The variance of component 1 (88.6%) explained the differences between PDRS11 and PDS5 (Fig. 4e), which had a very high accuracy (100%) (Fig. 4h). Ninety-two exhibited significant differences between PDRS11 and PDRS5 groups (*P* < 0.05). Seventy-five metabolites with VIP > 1 were significantly contributed to the separation of PDRS11 from PDRS5 (Fig. 4f, g).

### Fold changes of important metabolites with a VIP value > 1 in PDRS, PDRS5 and PDRS11 embryos

**Fig. 5:**
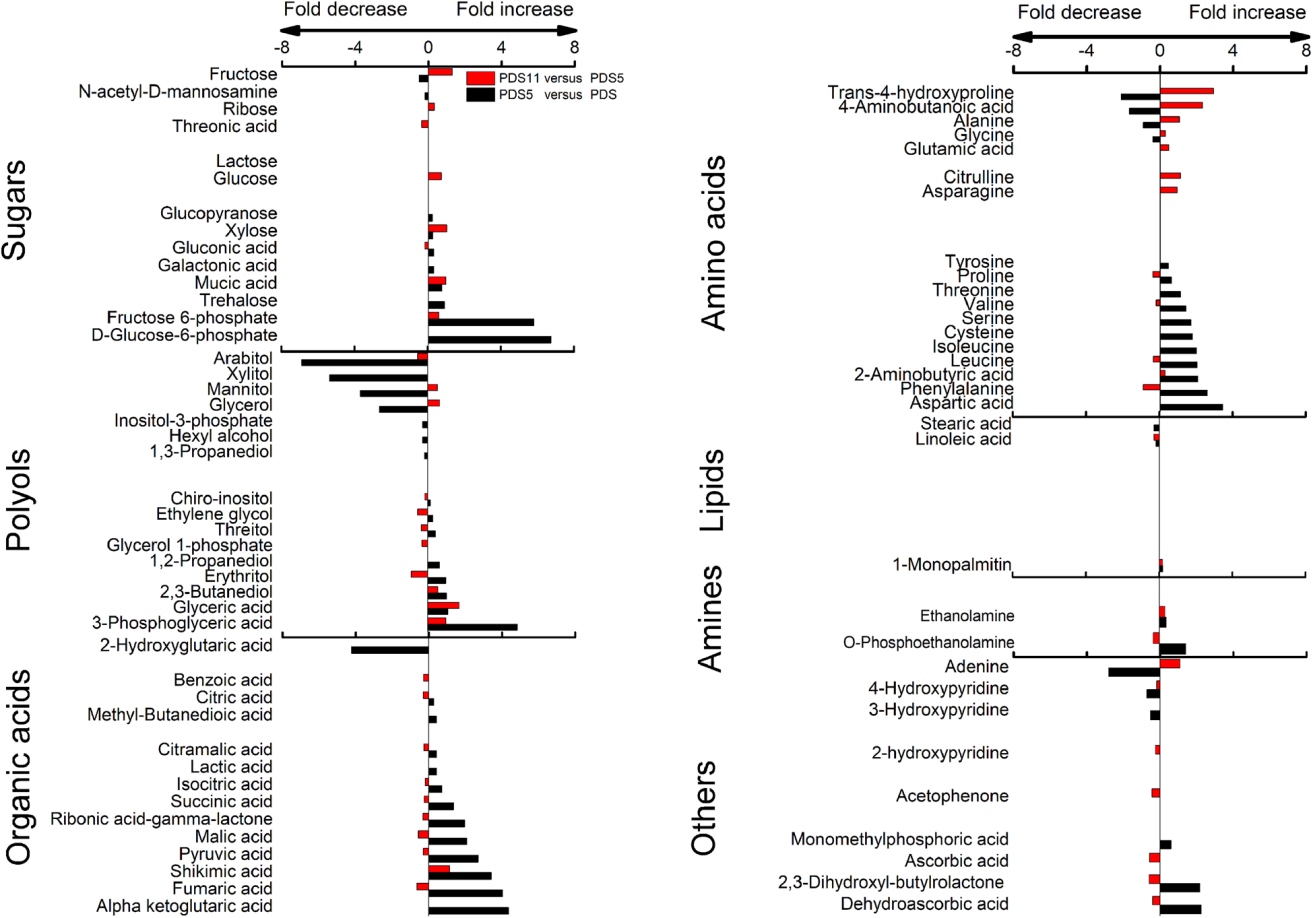
Changes in the contents of important metabolites with VIP value > 1 in the embryos of PDRS. The bar charts indicate the result for the log_2_ transformed. PDRS, seeds released from primary dormancy; PDRS5, 5-day incubated seeds released from primary dormancy and PDRS11, 11-day incubated seeds released from primary dormancy. Value bars facing the right of each section indicate the fold-increased content. Value bars facing the left of each section indicate the fold-decreased content

83% of the metabolites with VIP > 1 (including the majority of sugars, organic acids and amino acids) accumulated from days 0 to 5 (Fig. 5). 73% of the metabolites with VIP > 1 (including the majority of organic acids and amino acids) reduced substantially from days 5 to 11. Fructose 6-phosphate, inositol-3-phosphate, 3-phosphoglyceric and D-glucose-6-phosphate contents showed the most decrease, with decreasing 409-, 75-, 58- and 41-fold, respectively (Fig. 5). There was a 2–6 fold increase in the levels of xylose, glucose, glucopyranose, fructose and trehalose from days 0 to 5 and a further 1–3 fold continual increase from days 5 to 11 (Fig. 5).

### Partial least squares discriminant analysis of metabolites in PDS, PDS5 and PDS11 embryos

The data were clearly separated into two groups, resulting score plot showing a strong separation between PDS5 and PDS (Fig. 4i). Contents of seventy-seven metabolites exhibited significant differences between PDS5 and PDS (*P* < 0.05). Sixty-four metabolites with VIP > 1 significantly contributed to the separation of PDS5 from PDS (Fig. 4j, k). The flexibility of the model was evaluated by explained variance R^2^ = 0.99 and accuracy in prediction Q^2^ = 0.98 (Fig. 4l).

The variance of component 1 (59.4%) explained the differences between PDS11 and PDS5 (Fig. 4m). Contents of sixty-eight metabolites of the identified metabolites exhibited significant differences between PDS11 and PDS5 (*P* < 0.05). Fifty-two metabolites with VIP > 1 significantly contributed to the separation of PDS11 from PDS5 (Fig. 4n, o). The model R^2^ and Q^2^ scores were 0.97 and 0.95, respectively (Fig. 4p).

### Fold changes of important metabolites with a VIP value > 1 in PDS, PDS5 and PDS11 embryos

**Fig. 6:**
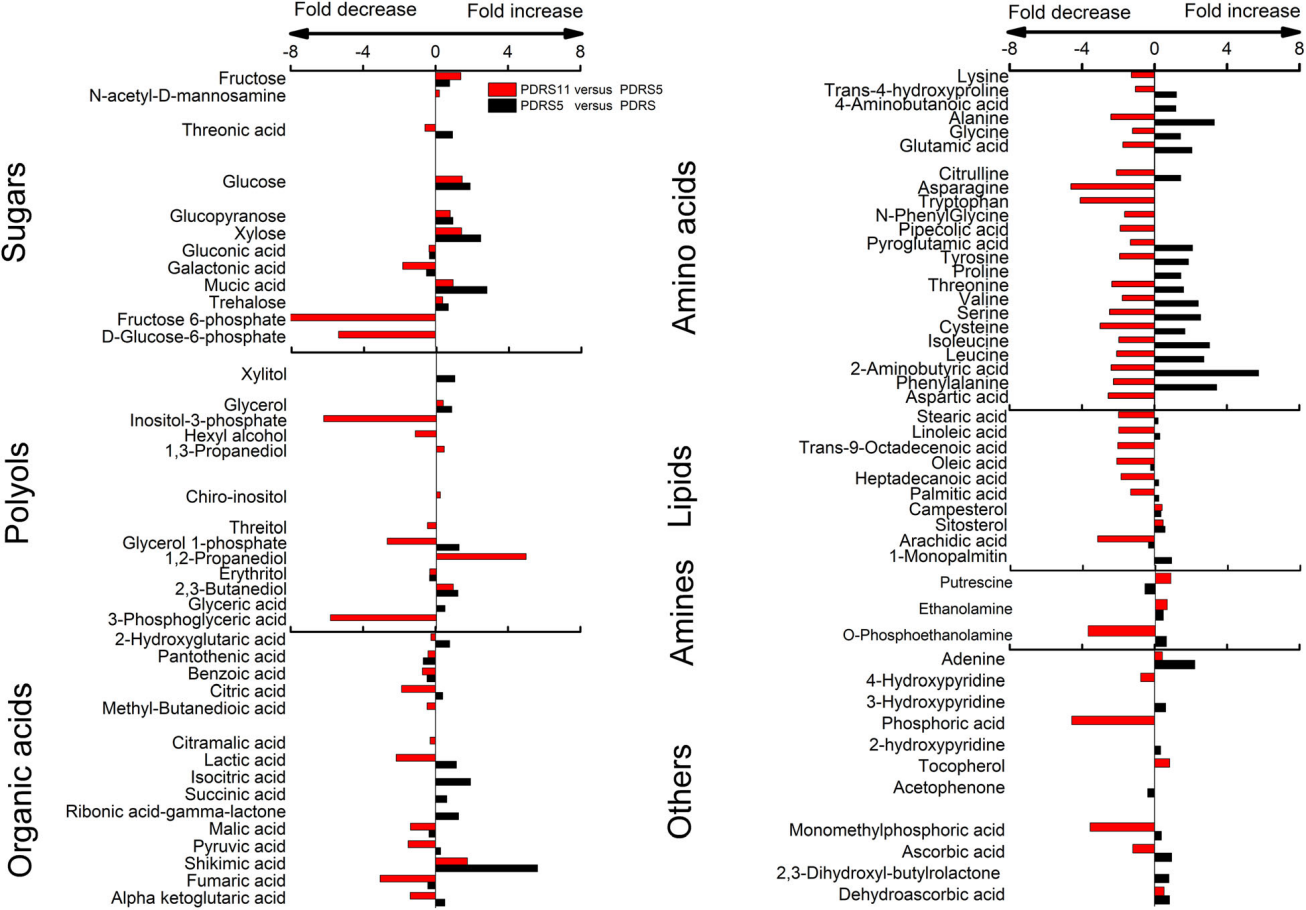
Changes in the contents of important metabolites with VIP value > 1 in the embryos of PDS. The bar charts indicate the result for the log_2_ transformed. PDS, primary dormant seeds; PDS5, 5-day incubated primary dormant seeds and PDS11, 11-day incubated primary dormant seeds. Value bars facing the right of each section indicate the fold-increased contents. Value bars facing the left of each section indicate the fold-decreased contents

The contents of 70% metabolites with VIP > 1 (including the majority of sugars, organic acids and amino acids) accumulated from days 0 to 5. Contents of D-glucose-6-phosphate, fructose 6-phosphate, 3-phosphoglyceric acid, oxoglutaric acid and fumaric acid showed the apparent changes, increasing 104-, 55-, 30-, 21- and 16-fold, respectively (Fig. 6). The levels of these metabolites with VIP > 1 displayed relatively small changes from days 5 to 11.

### The main altered metabolic pathways in PDRS embryos

**Fig. 7:**
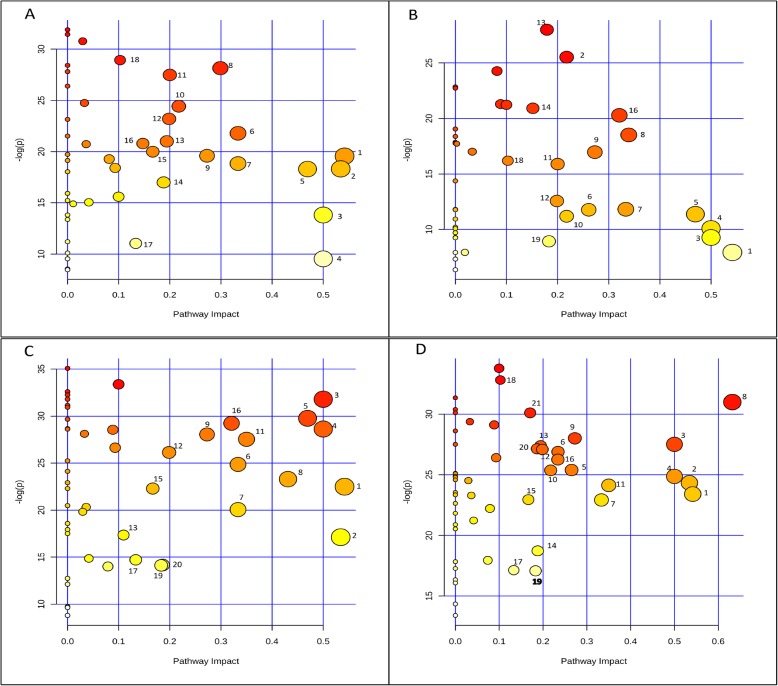
Metabolome view of the altered metabolic pathways in the embryos between (**a**) PDRS5 and PDRS, (**b**) PDRS11 and PDRS5, (**c**) PDS5 and PDS, (**d**) PDS11 and PDS5. PDRS, seeds released from primary dormancy; PDRS5, 5-day incubated seeds released from primary dormancy; PDRS11, 11-day incubated seeds released from primary dormancy; PDS, primary dormant seeds; PDS5, 5-day incubated primary dormant seeds and PDS11, 11-day incubated primary dormant seeds. Carbohydrate metabolisms (5, Glyoxylate and dicarboxylate metabolism; 6, Citrate cycle; 10, Fructose and mannose metabolism; 12, Pyruvate metabolism; 13, Glycolysis or Gluconeogenesis, 18, Amino sugar and nucleotide sugar metabolism; 14, Pentose phosphate pathway; 19, Ascorbate and aldarate metabolism). Amino acid metabolisms (1, beta-Alanine metabolism; 2, Glycine, serine and threonine metabolism; 3, Phenylalanine metabolism; 8, Alanine, aspartate and glutamate metabolism; 9, Tyrosine metabolism; 16, Arginine and proline metabolism; 21, Tryptophan metabolism). Energy metabolisms (15, Methane metabolism; 17, Sulfur metabolism). Lipid metabolism (7, Sphingolipid metabolism; 20, Glycerophospholipid metabolism). Metabolisms of cofactors and vitamins (11, Pantothenate and CoA biosynthesis). Biosynthesis of other secondary metbaolites (4, Isoquinoline alkaloid biosynthesis)

Those pathways located on the top right corner of the ‘metabolome view’ are the main altered metabolic pathways (Fig. 7). From days 0 to 5, the main altered metabolic pathways included amino acid metabolisms (glycine, serine and threonine metabolism, alanine, aspartate and glutamate metabolism, arginine and proline metabolism and tyrosine metabolism), carbohydrate metabolism (glyoxylate and dicarboxylate metabolism, pyruvate metabolism, TCA cycle and glycolysis) and the metabolism of cofactors and vitamins (pantothenate and CoA biosynthesis) and glycerophospholipid metabolism (Fig. 7a).

From days 5 to 11, the following metabolic pathways were mainly altered, including amino acid metabolism (alanine, aspartate and glutamate metabolism, beta-alanine metabolism, glycine, serine and threonine metabolism and arginine and proline metabolism), carbohydrate metabolism (citrate cycle, pyruvate metabolism, glycolysis and pentose phosphate pathway), lipid metabolism (sphingolipid metabolism) and the metabolism of cofactors and vitamins (pantothenate and CoA biosynthesis) (Fig. 7b).

### The main altered metabolic pathways in PDS embryos

With the exception of glycerophospholipid metabolism, those metabolic pathways significantly changed in PDRS embryos also displayed significant alternation in PDS embryos from days 0 to 5 (Fig. 7c). In addition, pentose phosphate pathway also showed major alternation. The pronouncedly altered metabolic pathways from days 0 to 5 significantly changed from days 5 to 11, except for glycine, serine and threonine metabolism and pyruvate metabolism (Fig. 7d).

### Comparative metabolic pathway analysis between PDRS and PDS

**Fig. 8:**
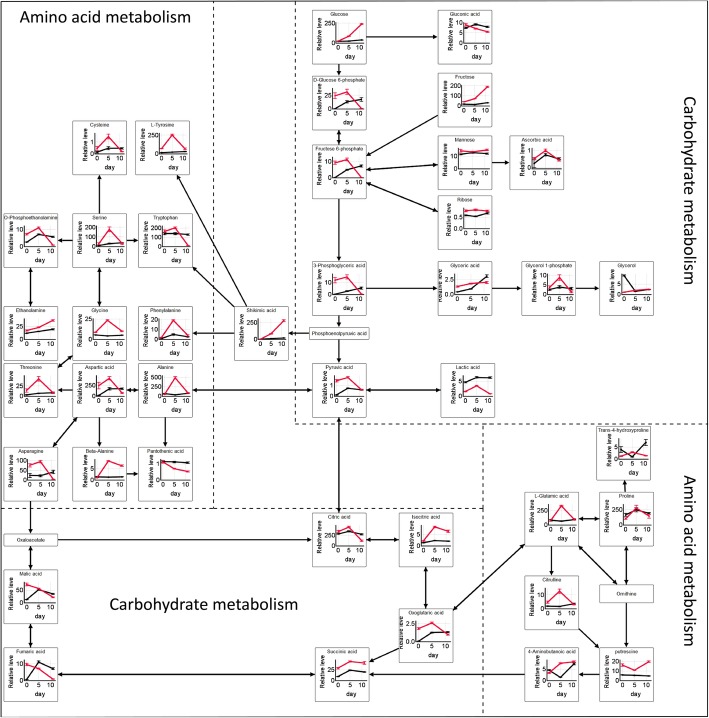
Integrative overview of the major metabolic changes in the embryos of PDRS (seeds released from primary dormancy) and PDS (primary dormant seeds) under germination conditions. Red and Black colors indicate PDRS and PDS, respectively

From days 0 to 5, there was an increased activity of carbohydrate metabolism and amino acid metabolism in PDRS embryos and a similar but less intense increase in PDS embryos (Fig. 8). From days 5 to 11, most intermediates of carbohydrate metabolism and amino acid metabolism in PDRS embryos decreased substantially. In contrast, there were minor changes in these metabolites (including sugars, organic acids and amino acids) in PDS embryos.

## Discussion

The contents of 76 and 64% metabolites substantially increased in PDRS and PDS embryos from days 0 to 5, respectively, implying that biosynthetic metabolic processes are more predominant. Specifically, carbohydrate metabolism (i.e., glyoxylate and dicarboxylate metabolism, TCA cycle, pyruvate metabolism and glycolysis) and amino acids metabolisms (i.e., glycine, serine and threonine metabolism, alanine, aspartate and glutamate metabolism, arginine and proline metabolism and tyrosine metabolism) were mainly stimulated. Other studies also showed that both dormant and nondormant seeds have upregulated genes after 24 h of water imbibition [32]. Respiration and energy production could proceed in both dormant and nondormant seeds [33]. The activation of metabolic pathways depends on water entry. The increase in the levels of most metabolites in PDS embryos after 5 days of incubation is most likely associated with rapid initial water uptake by dry seeds. It has also been documented that dramatic water uptake in Korean pine seeds occurred from days 0 to 5 under germination conditions (Fig. 1c). The levels of carbohydrate metabolic pathways intermediates in both PDS5 and PDS11 embryos were significant lower compared with PDRS5 embryos, implying that the degree of increasing activities of carbohydrate metabolism in PDS embryos is much lesser. The attenuated carbohydrate metabolism could be associated with the prevention of storage reserve degradation. It has also been reported that the expression of genes involved in carbohydrate degradation gradually declined following transfer of dormant *Euphorbia esula* seeds to germination conditions for 21 days of incubation [34].

De novo protein synthesis is required for germination. However, the contents of free amino acids are too low to support protein synthesis during germination [30], therefore, storage protein is degraded into amino acids. The proteins in embryo are first degraded and then their components are used locally [30]. Although the activities of amino acids metabolisms increased in both PDS and PDRS embryos from days 0 to 5, the increasing degree is less in PDS embryos. Therefore, it is plausible that amino acids biosynthesis pathways are highly repressed in PDS embryos compared with PDRS embryos. The expression levels of genes encoding protein degradation, the abundances of important components of amino acid and protein metabolisms progressively declined as increasing imbibition time of dormant seeds [21, 34]. In contrast, there is an interconversion between amino acids once seed dormancy is released. De novo synthesis pathways of amino acids can also be induced.

Korean pine seeds contain greater than 60% fat, and the majority of fat is located in megagametophyte [35]. Oxygen is required for β-oxidation of fatty acids to produce acetyl-CoA. Acetyl-CoA is then converted to sucrose through the glyoxylate cycle and gluconeogenesis [36]. Lactate level was lower in PDRS embryos than that in PDS embryos from days 5 to 11, indicating that the amounts of oxygen are relatively higher in PDRS embryos. However, after cracking seed coats, the respiration rates of moist chilled seeds were higher compared with intact moist chilled seeds. It can be inferred that the levels of oxygen in seeds with released primary dormancy is still insufficient. Oxygen consumption in the embryos of PDRS may be reduced by diminishing glycolysis and TCA cycle. Therefore, more oxygen may be supplied to megagametophyte for β-oxidation of fatty acids, leading to the accumulation of sucrose. Sugars were then transported throughout the embryo, where it supports embryo growth and development. The continual accumulation of sugars (xylose, glucose, glucopyranose, trehalose, fructose and sucrose) in the embryos of PDRS from days 0 to 11 may also support this hypothesis. In *Pinus lamberdaea* seeds released from primary dormancy, sucrose levels also constantly accumulated during germination [37]. The accumulation of osmotically active substances (sugars and amino acids) results in a further increase in water uptake [38]. The substantial increase in the levels of most sugars may cause seed to absorb water more rapidly, leading to testa rupture. However, this conclusion needs to be further demonstrated by other experiments.

After cracking seed coat, the respiration rates of PDS increased rapidly, indicating that seed coat restricts germination by limiting oxygen uptake. The maintenance of higher lactate levels in PDS embryo compared with PDRS embryos further documented that the availability of oxygen is limited in PDS embryos. In addition, the levels of carbohydrate metabolism (i.e., TCA cycle, glycolysis and pentose phosphate pathway) intermediates in PDS11 were significant higher than that in PDRS11. These data indicate that when oxygen is not sufficient in PDS embryo, glycolysis and TCA cycle still occur in a relatively high rate, consuming large amounts of oxygen. Thus, the availability of oxygen in PDS megagametophytes might be reduced, leading to retarded β-oxidation of fatty acids. It can also be found that only the levels of xylose, glucose and lactose in PDS embryos increased slightly from days 0 to 11. Furthermore, the levels of 14 out of 16 sugars in PDS embryos were significantly lower compared with PDRS embryos. After transfer of primary dormant *Pinus lamberdaea* seeds to germination conditions, sucrose levels initially increased and then subsequently decreased [37]. The sugar contents in PDS embryos may be too low to cause water absorption and seed coat rupture. Therefore, seed primary dormancy was maintained. PDS cannot make corresponding and rapid adjustments from days 5 to 11 (i.e., reducing glycolysis and TCA cycle) to respond to the surrounding hypoxic environment, whereas PDRS can. However, it is necessary to further elucidate the relationship between primary dormancy maintenance and oxygen and sugar distribution in the embryo and megagametophyte of Korean pine seeds.

The levels of amino acids participating in alanine, aspartate and glutamate metabolism, beta-alanine metabolism, glycine, serine and threonine metabolism and arginine and proline metabolism in PDRS embryos substantially decreased from days 5 to 11. De novo protein synthesis occurred during early phase II of *Arabidopsis* seed germination [39]. While, several amino acids in PDS embryos slightly decreased from days 5 to 11, suggesting that de novo synthesis of protein was blocked. The expression levels of genes related to protein synthesis declined in imbibed dormant seeds [40]. Dormant seeds are enriched for genes related to repressed translation capacity [39–41]. Glutamate, aspartate, asparagine and proline were the major amino acids in both PDS and PDRS embryos. These amino acids are also dominant in *Pinus taeda* and *Pinus banksiana* seeds [42, 43]. Glutamine and asparagine have high nitrogen/carbon ratio and are particularly suitable for the storage of N [44], and are frequently used as transport compounds transporting nitrogen to the rapidly growing tissues [43, 45]. A 24-fold decrease of asparagine occurred in PDRS embryos from days 5 to 11, but there was a 2 fold increase of asparagine in PDS embryos, implicating that the utilization of amino acids is likely less intense in PDS embryos. Polyamines (putrescine, spermidine and spermine) can activate the occurrence of protein synthesis [24]. Putrescine accumulated rapidly in PDRS embryos but declined gradually in PDS embryos from days 5 to 11, implicating that the capacity of protein synthesis is lower in PDS embryos. This change in amino acid metabolism in PDRS embryos from days 0 to 11 follows similar patterns as reported previously [18].

## Conclusions

The embryos of PDRS exhibited different patterns of metabolite profiles between the first 5 days and the last 6 days of germination sensu stricto. From days 0 to 5, there was a rapid accumulation of 76% of metabolites in the embryos of PDRS, suggesting that biosynthetic metabolic processes are initiated. However, from days 5 to 11, the levels of 67% of metabolites substantially decreased. Especially, almost all amino acids and the glycolysis and TCA cycle intermediates displayed a relatively large reduction. The high efficient utilization of almost all examined amino acids in the embryos of PDRS may provide proteins for embryo growth. The attenuated glycolysis and TCA cycle may make oxygen consumption decrease, leading to the accumulation of sugars in the embryos of PDRS. The continual increase of sugars, especially sucrose, in the embryos of PDRS from days 0 to 11 document this speculation. The substantial increase in the levels of most sugars in the embryos of PDRS may cause seed to absorb water more rapidly, leading to seed coat rupture. From days 0 to 5, the levels of 64% of metabolites in the embryos of PDS increased, accompanied with a rapid water uptake. Moreover, the levels of these metabolites were not only further accumulated, but also lower compared with PDRS from days 5 to 11. These results indicate that biosynthetic metabolic processes are impeded in the embryos of PDS from days 0 to 5. Unlike PDRS embryos, almost all amino acids in PDS embryos did not exhibit a substantial decrease from days 5 to 11. Also, there was not a major decrease in the levels of metabolites involved mainly in glycolysis and TCA cycle, while some intermediates even increased. The inferences from this study might help in better understanding the seed dormancy mechanisms and establishing a simple and very efficient means to release seed dormancy.

## Methods

Two batches of seeds were used in the present study. Seeds collected in October 2013 were used for the determination of gas permeability and water permeability of seed coat. The presence or absence of a gas barrier is identified by determining the difference in respiration rate between the intact primary dormant seeds and seeds with cracked seed coats. By monitoring the changes in the masses of intact primary dormant seeds under germination conditions, we determined whether the seed coat interferes with water uptake. Seeds collected in 2014 were used for metabolomics analysis.

### Seed collection

Fresh Korean pine cones were collected from 30 trees aged about 50 years old in a Korean pine plantation in October 2013 and 2014, respectively, at the Qingyuan Forest CERN, CAS (Chinese Academy of Sciences), Northeast China (41°51.102′ N, 124°54.543′ E, 456–1116 m a.s.l.). The gymnosperm cones were opened to release seeds by using threshing machine. Then, these fresh seeds are dried indoors until seed water content is around 10%. Seed water content was determined by weighing fresh seeds, then drying these fresh seeds at 103 °C for 17 h and reweighing. These fresh dry seeds were then stored at − 20 °C until experiment.

### Determination of water permeability and gas permeability of seed coat

Seeds collected in October 2013 were immediately used for the determination of gas permeability and water permeability of seed coat. The respiration rates of intact primary dormant seeds and the seeds with cracked seed coats were determined. Every 25 seeds (intact primary dormant seeds or seeds with cracked seed coats) were placed in a 9-cm diameter Petri dish with eight layers of filter paper that was moistened with 12 ml deionized water. Each Petri dish was placed in a 1.3 L air-tight plastic box. At the same time, a beaker containing 10 ml NaOH (1 mol L^− 1^) solution was also put in plastic box to trap CO_2_. The plastic box was then incubated in light/dark (14/10 h) at alternating temperature regimes of 25/10 °C in a controlled growth chamber (MGC-450HP-2, Bluepard, Shanghai, China). This incubation temperature was selected according to the NFSSTC standards for the *Pinus* [46]. Photosynthetic photon flux density was about 120 μmol m^− 2^ s^− 1^ located at the top of Petri dishes. BaCl_2_ (1 mol L^− 1^) was used to precipitate CO_2_ absorbed by NaOH in the beaker. Then the remaining NaOH in the beaker was titrated with 1 mol L^− 1^ HCL. The volume of HCL consumed during the titration is used to calculate the amount of CO_2_ produced by seed respiration. Deionized water was added at any time during incubation to ensure suitable water conditions for seed respiration. The respiration rate was measured every day and expressed as μmol CO_2_ g^− 1^ min^− 1^. Three replicates were carried out separately for seed respiration.

Every 25 intact primary dormant dry seeds were placed in a 9-cm diameter Petri dish. Eight layers of filter paper moistened with 12 ml deionized water were placed in each Petri dish. Three Petri dishes were used as three replicates. The Petri dish was covered with plastic wrap to reduce moisture loss and then incubated under germination conditions. Every 24 h, seeds were removed from the filter paper, blotted dry, weighed to the nearest 0.1 mg and returned to the Petri dishes. The percentage of increase in seed mass was calculated using the following formula. The percentage of increase in seed mass = [(the masses of seeds incubated under germination conditions – the masses of primary dormant dry seeds)/ the masses of primary dormant dry seeds] × 100.

### Preparation of seeds that were used for metabolomics analysis

The seedlot collected in October 2014 was divided into two portions, one of which was immediately stored at -20 °C until examining the metabolism of primary dormancy. Another portion of the seedlot was used for moist chilling in early November. The specific procedure of moist chilling was as follows: seeds were first given a 7-day running water soak and then buried in soil at 50 cm depth for 6 months (November 2014–April 2015) in the Korean pine plantation [28].

After moist chilling, the germination capacity of moist chilled seeds was assessed to ascertain whether they had been released from primary dormancy. The seeds released from primary dormancy in the present study refer to those seeds treated with approximately 6 months of moist chilling. In late April 2015, fresh dry seeds were removed from − 20 °C storage conditions. The germination experiment was subsequently conducted with these seeds to determine whether the seeds are primary dormant status. The primary dormant seeds in the present study refer to those dry seeds treated with approximately 6 months of storage at − 20 °C. The seeds released from primary dormancy and primary dormant seeds were then used for metabolomics analysis.

Each germination test consisted of three replications of 20 seeds each. These 20 seeds were placed in a 9-cm diameter Petri dish with eight pieces of filter paper moistened with deionized water. Deionized water was added to Petri dish to ensure appropriate moisture required for seed germination. All dishes were wrapped with plastic film to reduce water loss and then incubated in light/dark (14/10 h) at alternating temperature regimes of 25/10 °C in a controlled growth chamber (MGC-450HP-2, Bluepard, Shanghai, China). Photosynthetic photon flux density was about 120 μmol m^− 2^ s^− 1^ located at the top of Petri dishes. Germination was tested every two days for 6 weeks and was considered to be completed when radicle protrusion was greater than 2 mm [47] . At the end of the germination test, the seeds failing to complete germination were cut to test the seed embryo viability with a tetrazolium method.

### Experimental design of metabolomics analysis

Two types of seeds including PDRS (seeds released from primary dormancy) and PDS (primary dormant seeds) were used for metabolomics analysis in this study. PDRS or PDS were placed in a 9-cm diameter Petri dishes with eight layers of filter paper moistened using 10 ml of distilled water for water imbibition and then kept in light/dark (14/10 h) at alternating temperature regimes of 25/10 °C in order to induce germination. Embryos (20 per replicate and 3 biological replicates per seed type) were excised from seeds at 0, 5, and 11 days after germination. The radicle of PDRS protruded the seed coat until 11 days of imbibition under a germination-inductive condition. This stage corresponds to the germination sensu stricto (i.e., none of the seeds showed visible germination at this stage). However, PDS cannot germinate during this period, implying that the primary dormancy is still maintained. 5-day incubated PDRS, 11-day incubated PDRS, 5-day incubated PDS and 11-day incubated PDS were subsequently abbreviated as PDRS5, PDRS11, PDS5 and PDS11, respectively. At each seeds sampling time point, the embryos were dissected from the rest of the seed structure, immediately frozen in liquid N_2_ and pulverized in liquid N_2_, lyophilized and stored at − 20 °C until metabolite analysis.

### Sample preparation

The 100 mg embryo powder was put into a 2 mL Eppendorf tube and extracted with 1.5 mL of 80% methanol. In order to adequately extract the metabolites, the sample was first vortexed for 5 min and then centrifuged at 20598 x g for 10 min. After that, 800 μL of the supernatant was removed into another 1.5 mL Eppendorf tube and lyophilized for 10 h. Then, 100 μL of methoxyamine solution (20 mg mL^− 1^) preparing with pyridine was added to the Eppendorf tube to dissolve the dry residue. This solution sample was then incubated in a water bath for 90 min at 37 °C to perform the oximation reaction [49, 50]. Subsequently, 80 μL of MSTFA (N-Methy-N-(trimethyl-silyl) trifluoroacetamide) was added to the sample and incubated in a water bath for 60 min at 37 °C to conduct the silylation reaction [49, 50]. Then, 200 μL of supernatant was removed into a sample vial and used for GC-MS (gas chromatography-mass spectrometry) analysis.

### GC-MS analysis

A QP 2010 GC-MS equipped with an AOC-20i automatic injector (Shimadzu, Japan) was used in the present study. Chromatographic separation of metabolites was accomplished on a 30 m × 0.25 mm × 0.25 μm DB-5 MS column (J&W Scientific, Folsom, CA, USA). The injection temperature was set at 300 °C. Helium was used as a carrier gas. The constant flow of the carrier gas was set as 40 cm second^− 1^. The injection volume was 1 μL. The split ratio was set as 10:1. In order to achieve ionization of the metabolites, the electron impact model at 70 eV was used. The temperature of the interface was 280 °C. The temperature of the ion source was set at 230 °C. The mass spectra scan scope ranged from 33 to 500 m z^− 1^. The scan speed was 5 scans second^− 1^. The solvent cut time was 5.7 min. The column temperature was maintained at 70 °C for the first 3 min and then increased to 310 °C at the rate of 5 °C minute^− 1^. The 310 °C was maintained for 5 min.

Data processing.

A pseudotargeted method was established based on non-targeted GC-MS data of pooled QC sample. In brief, the acquired QC data was imported as netCDF format into AMDIS (automated mass spectral deconvolution and identification system) 2.62 (NIST, USA) for peak deconvolution, alignment and detection. After that, peak intensity and retention time was obtained and then the characteristic ions of the compounds to be detected were screened using chromTOF 4.43 (LECO, USA). Notably, a triple concentrated QC sample was used for qualitative analysis to cover the low abundant compounds. In the end, grouping was obtained by calculating the retention time interval of two successive peaks. A table for selected ion monitoring was generated with multiple groups containing information on the time range and the characteristic ions of the compounds to be detected.

After data acquisition, a quantitative table containing the retention time and the characteristic ions of all detected compounds and the internal standard was exported as a excel file using GCMS Postrun Analysis (GCMSsolution Version 2.70, Shimadzu Corporation, Japan). The peak area of all detected compounds was normalized to that of the internal standard. Methanol dissolved tridecanoic acid (0.4 μg/mL) was used as an internal standard for data normalization. Only the detected compounds with RSDs (relative standard deviations) less than 20% in all QC samples were included in the final data matrix.

### Statistical analysis

The germination percentage was defined as the number of seeds completing germination/total number of viable seeds × 100%. One-way ANOVA (analysis of variance) following LSD (least—significant difference) post hoc analysis were used to test for significant differences in respiration rate, seed mass and germination percentage between incubation times. The percentage of metabolites with increased (decreased) levels was calculated as the numbers of metabolites with increased (decreased) levels/total numbers of identified metabolites × 100%. A paired sample t-test was used to determine the difference in the levels of metabolites between PDRS5 and PDRS, PDRS11 and PDRS5, PDS5 and PDS, PDS11 and PDS5, respectively. A principal component analysis (PCA) was employed to summarize the systematic alteration of 6 samples (PDS, PDS5, PDS11, PDRS, PDRS5 and PDRS11) using SIMCA-P 11 software (Sweden). In order to visualize the patterns of change in the differential metabolites, HCA (hierarchical cluster analysis) was conducted with MetaboAnalyst. Heatmaps were based on normalized data using autoscale features for standardization, clustering with average linkage algorithm, distance measure with Pearson correlation coefficients. The heatmap was then divided into four groups to clearly show the relationships and trends among the differential metabolites in the six samples. PLS-DA (Partial least squares discriminant analysis) was then used to identify the differentially expressed metabolites contributing to the separation of each of the four pairs of samples with MetaboAnalyst. All metabolite data were UV (unit-variance) scaled before performing PLS-DA. In UV-scaling, each variable was mean-centered and divided by the standard deviation. A total of four partial least squares discriminant analyses were conducted. Specifically, those differentially expressed metabolites were identified by inspecting loadings plots from PLS-DA. Only those metabolites with VIP (variable importance in the projection) > 1 and significant changes (*P* < 0.05) in content were considered to have significant contributions to the classification of each of the four pairs of samples [48, 51, 52]. These four pairs of samples are as follows: PDRS5 vs PDRS, PDRS11 vs PDRS5, PDS5 vs PDS and PDS11 vs PDS5. Fold changes of the metabolites with VIP > 1 between each of the four pairs of samples were also calculated. Fold change was calculated as the ratio between two group means and then log_2_ transformed. Specifically, they were calculated by the following formulas: log_2_^(PDRS5/PDRS)^, log_2_^(PDRS11/PDRS5)^, log_2_^(PDS5/PDS)^ and log_2_^(PDS11/PDS5)^. The calculation of fold changes was conducted with MetaboAnalyst. Those metabolites with VIP > 1 were then subsequently subjected to metabolic pathway analysis to identify and visualize the significantly altered metabolic pathways between PDRS5 vs PDRS, PDRS11 vs PDRS5, PDS5 vs PDS and PDS11 vs PDS5. The pathway enrichment analysis and pathway topological analysis in the MetaboAnalyst web tool were used together to perform metabolic pathway analysis [53]. The *P* value calculated with the ‘Global Test’ algorithm in pathway enrichment analysis was used to indicate the significance of enriched metabolic pathways [54]. The impact value calculated with the ‘Relative-Betweenness Centrality’ algorithm in pathway topological analysis was used to estimate the relative importance of metabolic pathways [53]. The impact-value threshold was set to 0.1 to identify the most relevant metabolic pathways [55, 56]. Finally, the result of the metabolic pathway analysis (metabolome view) was rendered in a graph format. The *P* value of each metabolic pathway was log-transformed and then set as the Y-axis and the pathway impact value was set as the X-axis. Each node in a graph represents a metabolic pathway. The node color indicates the *P* value of each metabolic pathway, while the node radius indicates the impact value of each metabolic pathway [54]. Therefore, dark red, large circles located in the top right corner of the “metabolome view” represent the main altered pathways compared to the yellow, small circles located in the left of the graphs. The software VANTED was then used to visualize the pathway map of the significantly altered metabolites [57].

## Data Availability

The datasets used and/or analyzed during the current study are available from the authors on reasonable request (Jiaojun Zhu, jiaojunzhu@iae.ac.cn; Yuan Song, songyuan_in2000@yeah.net).

## Abbreviations

AMDIS: automated mass spectral deconvolution and identification system
ANOVA: analysis of variance
ATP: adenosine triphosphate
CAS: Chinese Academy of Sciences
GC-MS: gas chromatography-mass spectrometry
HCA: hierarchical cluster analysis
LSD: least-significant difference
MBKPFs: mixed broadleaf Koran pine (*Pinus koraiensis* Sieb. et Zucc.) forests; MSTFA N-Methy-N-(trimethyl-silyl) trifluoroacetamide
PCA: principal component analysis
PDS: primary dormant seeds
PDS5: 5-day incubated primary dormant seeds
PDS11: 11-day incubated primary dormant seeds
PDRS: seeds released from primary dormancy
PDRS5: 5-day incubated seeds released from primary dormancy
PDRS11: 11-day incubated seeds released from primary dormancy
PLS-DA: partial least squares discriminant analysis
RSDs: relative standard deviations
TCA: tricarboxylic acid cycle
UV: unit-variance
VIP: variable importance in the projection

## References

[1] Zhu JJ, Mao ZH, Hu LL, Zhang JX (2007). Plant diversity of secondary forests in response to anthropogenic disturbance levels in montane regions of northeastern China. J For Res.

[2] Tian Y, Wu JG, Kou XJ, Wang TM, Mou P, Ge JP (2009). Spatiotemporal pattern and major causes of the Amur tiger population dynamics. Biodivers Sci.

[3] Wang YJ (1995). Korean pine forest.

[4] Li JW, Guo QX, Li P (1995). Study on the cutover land of the hardwood-Korean pine forest for reproduction in the Xiaoxingan Mountains of Northeast China. J Northeast Fore Univ.

[5] Li YB, Mou P, Wang TM, Ge JP (2012). Evaluation of regeneration potential of *Pinus koraiensis* in mixed pine-hardwood forests in the Xiao Xing’an mountains, China. J For Res.

[6] Song Y, Zhu JJ, Yan QL, Wang GC (2018). Korean pine seed: linking changes in dormancy to germination in the 2 years following. Forestry..

[7] Finch-Savage WE, Leubner-Metzger G (2006). Seed dormancy and the control of germination. New Phytol.

[8] Baskin CC, Baskin JM (2014). Seeds: ecology, biogeography, and evolution of dormancy and germination 2nd ed.

[9] Baskin CC, Thompson KM, Baskin J (2006). Mistakes in germination ecology and how to avoid them. Seed Sci Res.

[10] Bewley JD (1997). Seed germination and dormancy. Plant Cell.

[11] Rajjou L, Duval M, Gallardo K, Catusse J, Bally J, Job C, Job D (2012). Seed germination and vigor. Annu Rev Plant Biol.

[12] Ribeiro PR, Willems LAJ, Mutimawurugo MC, Fernandez LG, Castro RDD, Ligterink W, Hilhorst HWM (2015). Metabolite profiling of *Ricinus communis* germination at different temperatures provides new insights into thermo-mediated requirements for successful seedling establishment. Plant Sci.

[13] Chibani K, Ali-Rachedi S, Job C, Job D, Jullien M, Grappin P (2006). Proteomic analysis of seed dormancy in Arabidopsis. Plant Physiol.

[14] Downie B, Bewley JD (2000). Soluble sugar content of white spruce (*Picea glauca*) seeds during and after germination. Physiol Plant.

[15] Fei HM, Ferhatoglu Y, Tsang E, Huang DQ, Cutler AJ (2009). Metabolic and hormonal processes associated with the induction of secondary dormancy in *Brassica napus* seeds. Botany.

[16] Lewak S (2011). Metabolic control of embryonic dormancy in apple seed: seven decades of research. Acta Physiol Plant.

[17] Weckwerth W (2010). Metabolomics: an integral technique in systems biology. Bioanalysis..

[18] Das A, Kim DW, Khadka P, Rakwal R, Rohila JS (2017). Unraveling key metabolomic alterations in wheat embryos derived from freshly harvested and water-imbibed seeds of two wheat cultivars with contrasting dormancy status. Front Plant Sci.

[19] Gao F, Jordan MC, Ayele BT (2012). Transcriptional programs regulating seed dormancy and its release by after-ripening in common wheat (*Triticum aestivum* L.). Plant Biotechnol J.

[20] Xia Q, Maharajah P, Cueff G, Rajjou L, Prodhomme D, Gibon Y, Bailly C, Corbineau F, Meimoun P, El-Maarouf-Bouteau H (2018). Integrating proteomics and enzymatic profiling to decipher seed metabolism affected by temperature in seed dormancy and germination. Plant Sci.

[21] Arc E, Chibani K, Grappin P, Jullien M, Godin B, Cueff G, Valot B, Balliau T, Job D, Rajjou L (2012). Cold stratification and exogenous nitrates entail similar functional proteome adjustments during *Arabidopsis* seed dormancy release. J Proteome Res.

[22] Hance BA, Bevington JM (1992). Changes in protein synthesis during stratification and dormancy release in embryos of sugar maple (*Acer saccharum*). Physiol Plant.

[23] Noland TL, Murphy JB (1986). Protein synthesis and aminopeptidase activity in dormant sugar pine seeds during stratification and warm incubation. J Plant Physiol.

[24] Szczotka Z, Pawłowski T, Krawiarz K (2003). Proteins and polyamines during dormancy breaking of European beech (*Fagus sylvatica* L.) seeds. Acta Physiol Plant.

[25] Murphy JB, Noland TL (1982). Temperature effects on oxidative metabolism of dormant sugar pine seeds. Plant Physiol.

[26] Einali AR, Sadeghipour HR (2007). Alleviation of dormancy in walnut kernels by moist chilling is independent from storage protein mobilization. Tree Physiol.

[27] Xin HW, Lai L, Lai GH (1991). Physiological and biochemical studies on seed dormancy of *Pinus koraiensis*-effect of carbohydrates on germination of excised embryos and changes of carbohydrates in seeds during stratification. J Yuzhou Univ.

[28] Song Y, Zhu JJ (2016). How does moist cold stratification under field conditions affect the dormancy release of Korean pine seed (*Pinus koraiensis*)?. Seed Sci Technol.

[29] Mao ZJ, Yuan XY, Zu YG, Zhao GY (2003). Study the seed morphological characteristics and the seed coat microstructure of *Pinus sibirica* and *P koraiensis*. Sci Silvae Sin.

[30] Bewley JD, Bradford K, Hilhorst H, Nonogaki H (2013). Seeds: physiology of development, germination and dormancy.

[31] Oracz K, El-Maarouf-Bouteau H, Bogatek R, Corbineau F, Bailly C (2008). Release of sunflower seed dormancy by cyanide: cross-talk with ethylene signalling pathway. J Exp Bot.

[32] Yazdanpanah F, Hanson J, Hilhorst H, Bentsink L (2017). Differentially expressed genes during the imbibition of dormant and after-ripened seeds-a reverse genetics approach. BMC Plant Biol.

[33] Preston J, Tatematsu K, Kanno Y, Hobo T, Kimura M, Jikumaru Y, Yano R, Kamiya Y, Nambara E (2009). Temporal expression patterns of hormone metabolism genes during imbibition of *Arabidopsis thaliana* seeds: a comparative study on dormant and non-dormant accessions. Plant Cell Physiol.

[34] Chao WS, Doğramaci M, Anderson JV, Foley ME, Horvath DP (2014). The resemblance and disparity of gene expression in dormant and non-dormant seeds and crown buds of leafy spurge (*Euphorbia esula*). BMC Plant Biol.

[35] Wu X, Liu Y, Gong J, Feng C, Wang X, Li J (2011). Diversities of morphological characteristics and constituents of *Pinus koraiensis* Sieb. Et Zucc. Seeds from Changbai Moutain and Xiaoxing’ anling areas [in Chinese]. Chem Ind For Prod.

[36] Eastmond PJ, Germain V, Lange PR, Bryce JH, Smith SM, Graham IA (2000). Postgerminative growth and lipid catabolism in oilseeds lacking the glyoxylate cycle. Proc Natl Acad Sci U S A.

[37] Murphy JB, Hammer MF (1988). Respiration and soluble sugar metabolism in sugar pine embryos. Physiol Plant.

[38] Bove J, Jullien M, Grappin P (2001). Functional genomics in the study of seed germination. Genome Biol.

[39] Rajjou L, Gallardo K, Debeaujon I, Vandekerckhove J, Job C, Job D (2004). The effect of α-amanitin on the Arabidopsis seed proteome highlights the distinct roles of stored and neosynthesized mRNAs during germination. Plant Physiol.

[40] Cadman CS, Toorop PE, Hilhorst HWM, Finch-Savage WE (2006). Gene expression profiles of Arabidopsis cvi seeds during dormancy cycling indicate a common underlying dormancy control mechanism. Plant J.

[41] Bassel GW, Lan H, Glaab E, Gibbs DJ, Gerjets T, Krasnogor N, Bonner AJ, Holdsworth MJ, Provart NJ (2011). Genome-wide network model capturing seed germination reveals coordinated regulation of plant cellular phase transitions. Proc Natl Acad Sci U S A.

[42] Ramaiah PK, Durzan DJ, Mia AJ (1971). Amino acids, soluble proteins, and isoenzyme patterns of peroxidase during the germination of jack pine. Can J Bot.

[43] King JE, Gifford DJ (1997). Amino acid utilization in seeds of loblolly pine during germination and early seedling growth (I. arginine and arginase activity). Plant Physiol.

[44] Bray CM (1983). Nitrogen metabolism in plants: nitrogen interconversions and transport during plant development.

[45] Avila C, Suárez MF, Gómez-Maldonado J, Francisco M, Cánovas FM (2001). Spatial and temporal expression of two cytosolic glutamine synthetase genes in scots pine: functional implications on nitrogen metabolism during early stages of conifer development. Plant J.

[46] National Forest Seed Standardization Technical Committee. Rules for forest tree seed testing. 1999.

[47] Bai Y, Thompson D, Broersma K (2004). Douglas fir and ponderosa pine seed dormancy as regulated by grassland seedbed conditions. J Range Manag.

[48] Zhao J, Hu C, Zeng J, Zhao Y, Zhang J, Chang Y, Li L, Zhao C, Lu X, Xu G (2014). Study of polar metabolites in tobacco from different geographical origins by using capillary electrophoresis–mass spectrometry. Metabolomics.

[49] Zhou J, Zhang L, Chang Y, Lu X, Zhu Z, Xu G (2012). Alteration of leaf metabolism in bt-transgenic rice (*Oryza sativa* L.) and its wild type under insecticide stress. J. Proteome Res.

[50] Zhao Y, Zhao C, Lu X, Zhou H, Li Y, Zhou J, Chang Y, Zhang J, Jin L, Lin F (2013). Investigation of the relationship between the metabolic profile of tobacco leaves in different planting regions and climate factors using a pseudotargeted method based on gas chromatography/mass spectrometry. J Proteome Res.

[51] Wu D, Cai S, Chen M, Ye L, Chen ZH, Zhang HT, Dai F, Wu FB, Zhang GP (2013). Tissue metabolic responses to salt stress in wild and cultivated barley. PLoS One.

[52] Ren W, Yin J, Gao W, Chen SH, Duan JL, Li TJ, Li NZ, Peng YY, Yin YL (2015). Metabolomics study of metabolic variations in enterotoxigenic *Escherichia coli-infected* piglets. RSC Adv.

[53] Xia J, Wishart DS (2010). MetPA: a web-based metabolomics tool for pathway anal-ysis and visualization. Bioinformatics..

[54] Shan L, Liao F, Jin H, Ye FS, Tong PJ, Xiao LW, Zhou J, Wu CL (2014). Plasma metabonomic profiling of lumbar disc herniation and its traditional Chinese medicine subtypes in patients by using gas chromatography coupled with mass spectrometry. Mol Biol Syst.

[55] Wang XJ, Yang B, Zhang AH, Sun H, Yan GL (2012). Potential drug targets on insomnia and intervention effects of Jujuboside a through metabolic pathway analysis as revealed by UPLC/ESI-SYNAPT-HDMS coupled with pattern recognition approach. J Proteome.

[56] González-Domínguez R, García-Barrera T, Gómez-Ariza JL (2015). Metabolite profiling for the identification of altered metabolic pathways in Alzheimer’s disease. J Pharm Biomed.

[57] Junker BH, Klukas C, Schreiber F (2006). VANTED: a system for advanced data analysis and visualization in the context of biological networks. BMC Bioinformatics.

